# Drug switching in the Netherlands: a cohort study of 20 active substances

**DOI:** 10.1186/s12913-020-05494-x

**Published:** 2020-07-13

**Authors:** Pieter J. Glerum, Marc Maliepaard, Vincent de Valk, David M. Burger, Kees Neef

**Affiliations:** 1 Medicines Evaluation Board, P.O. Box 8275, 3503 GB Utrecht, The Netherlands; 2 Department of Clinical Pharmacy & Toxicology, Maastricht University Medical Centre+, Maastricht, The Netherlands; 3 Department of Pharmacology and Toxicology, Radboud University Medical Centre, Nijmegen, The Netherlands; 4 National Health Care Institute, Diemen, The Netherlands; 5 Department of Pharmacy, Radboud University Medical Centre, Nijmegen, The Netherlands

**Keywords:** Drug switching, Generic substitution, Interchangeability, Brand-name drugs, Generic drugs

## Abstract

**Background:**

For a patient, drug switches are not desirable (either between a brand-name drug and a generic drug, or between two generic drugs of the same active substance). Research into the causes of drug switches, and related adverse drug reactions, is hampered by the absence of quantitative data on drug switches.

**Methods:**

We describe the frequency of drug switches in the Netherlands for a selection of active substances. A retrospective cohort study was conducted using the Drug Information System of the National Health Care Institute in the Netherlands. We studied the Dutch patient population from mid-2009 to 2016. The selection of active substances (*n* = 20) was made based on a report by Lareb, the Netherlands Pharmacovigilance Centre, on adverse drug reactions related to drug switching, and we used qualitative and quantitative descriptive analyses. A drug switch is defined as the replacement of a patient’s prescribed drug with a similar drug from a different manufacturer.

**Results:**

We identified 23.8 million drug switches on a total of 206 million (11.6%) similar drug dispenses. The frequency of drug switches demonstrated a yearly peak in the period from January to March. In some months, for atorvastatin, losartan, pantoprazole, and irbesartan, more than 60% of similar drug dispenses were drug switches. Most drug switches (80.3%) were between two generic drugs, and 0.12% of these involved a drug from a European parallel import. The proportion of drug switches between two brand-name drugs decreased from 14.5 to 5.53% during our study period, and of these, 86.5% involved a drug from a European parallel import.

**Conclusions:**

Drug switching is common in the Netherlands, and most of the drug switches we studied are between generic drugs. The observed annual peak of drug switches is most likely explained by a specific Dutch reimbursement policy. Not only are the data valuable as is, but they also serve as a first step towards elucidating the reasons for the occurrence of these drug switches. In addition, these data can be used to put into perspective the adverse drug reactions associated with drug switching.

## Background

The use of generic drugs is an important tool to reduce healthcare spending. For instance, in the United States (US), generic drugs cost as little as 6% of the price of brand-name drugs, mainly because of the lower cost of research, development, registration, and competition between drug companies [1, 2]. In the US, 90% of all dispenses are a generic drug [3]; however, the market penetration of generic drugs differs worldwide. In Japan, the market share of generic drugs was only approximately 23% at the end of 2012, but it is expected to increase [4]. Furthermore, the market share varies greatly in European countries, for example between 17% in Switzerland and 83% in the United Kingdom [5].

Although the use of generic drugs is financially desirable, these drugs are not always well received. A substantial proportion of physicians, pharmacists, and the general population have a negative perception of the quality, efficacy, and safety of generic drugs and drug switches [6]. In addition, adverse drug reactions (ADRs) associated with drug switches are regularly reported to Lareb, the Netherlands Pharmacovigilance Centre [7]. Thus, clinical discomfort is experienced with a drug switch, and are a clear downside of the use of generic drugs from the perspective of the patient. In this paper, we use the term drug switch for a switch between 2 similar drug products of the same active substance, which can either be between a brand-name drug and a generic drug or between two generic drugs.

Drug switches are thus not desirable, and studies should be conducted to determine how the frequency of drug switches can be kept low. However, before the reasons for drug switches can be explored, a first step should be to investigate the frequency of those switches. This is largely undocumented, apart from a recent effort of the US Food and Drug Administration (FDA) [8]. Therefore, we aim to study the frequency of drug switches and additionally explore some of the reasons that could influence this frequency. Furthermore, in a future study, we aim to refine previously mentioned analyses of switch-related ADRs, since the number of drug switches is missing from that analysis. Given this future aim, we limit our study to the 20 active substances described in the ADR report and focus on the Netherlands.

The Netherlands is a country with a large generic drug market share of 75.6%. Drugs are not prescribed by brand name, but by name of the active substance, and a generic drug is dispensed in 97% of cases, if a generic option is available [9]. A possible reason for the large market share is that health insurance companies in the Netherlands are authorized by law to select a drug product (either a generic or brand-name drug) eligible for reimbursement from a group of interchangeable drug products with the same active substance; this is known as the ‘Preference Policy’ [10]. Contracts between insurance companies and drug product manufacturers typically last 1 or 2 years, and the choice of drug product is predominantly based on price [11] and is thus most likely to be a generic drug. Preferred drug products change on a regular basis, and patients are forced to switch between them. Other reasons for drug switches can only be hypothesized, as no overview of the Dutch situation exists. These reasons could originate from any action of the insurer, wholesaler, pharmacy, prescriber, pharmacist, or patient; drug shortages; and patients changing healthcare insurance companies (4–10% of the Dutch population each year) [12–14]. Parallel imports from drug products can also play a role. A parallel import is allowed if a drug is registered in the European Union (EU) and deemed (virtually) identical to a drug registered in the Netherlands [15].

We studied drug switches in the claims database of the National Health Care Institute in the Netherlands (ZIN). By studying the extent of drug switches and the trends in their frequency in the Netherlands, the role of influencing factors such as the Preference Policy can be postulated.

### Aim of the study

The aim of this study is to describe the frequency of drug switches for a selection of active substances, in the Netherlands, in order to better understand generic drug use, and the Dutch process of drug switching and related influencing factors.

## Methods

We conducted a retrospective cohort study with qualitative and quantitative descriptive analyses. The cohort is 96% of the insured Dutch population, using a selection of 20 active substances. The study is reported in line with The Strengthening the Reporting of Observational Studies in Epidemiology (STROBE) guideline [16].

### Drug switches

Drug switches were obtained from the Drug Information System (GIP) of the ZIN. This database contains information on reimbursed drugs prescribed by general practitioners and specialists and dispensed by pharmacists or dispensing general practitioners. It does not include drugs dispensed within hospitals in the Netherlands.

A drug switch is defined as the replacement of a patient’s prescribed drug with a similar drug dispensed within the preceding 150 days, for the same active substance, same strength, and same route of administration, but with the drug product coming from a different manufacturer. The drug products before and after the switch could both be generic or brand-name drugs.

We allowed a 150-day difference between dispenses in our definition because in Dutch practice, drugs are usually dispensed for 90 days of treatment. We allowed a safety margin of 60 days extra to account for possible non-adherence and for early dispenses. If the difference between two dispenses was more than 150 days, we assumed that the patient had stopped and started a new treatment episode. Moreover, the date of the dispense was used as the best estimate of the moment when the patient actually experienced the drug switch.

We collected data to quantify the number of repeat dispenses using identical selection criteria, but with both dispenses of a drug product coming from the same manufacturer. We use the term consecutive dispenses for the sum of repeat dispenses and drug switches.

### Active substances

The selection of 20 active substances was based on a study by Lareb in 2017 that described active substances with more than 25 ADR reports associated with drug switching between 2006 and 2016 [7]. The following active substances are included in this study: atorvastatin, enalapril, esomeprazole, ethinylestradiol/levonorgestrel, irbesartan, levothyroxine, losartan, metformin, methotrexate, methylphenidate, metoprolol, omeprazole, pantoprazole, paroxetine, perindopril, rivastigmine, salbutamol, salmeterol/fluticasone, simvastatin, and venlafaxine. Of note, while the total number of active substances was 20, the total number of unique Anatomical Therapeutic Chemical (ATC) codes was 21. This is because methotrexate can be used as an antineoplastic agent (ATC: L01BA01) and as an immunosuppressant (L04AX03).

We defined the time frame of our study according to the maximum availability of data, which was 7.5 years from 01 June 2009 to 31 December 2016.

### Data analyses

Data were aggregated on a monthly basis, as this was expected to maximize to potential to study the influence of the Preference Policy.

We performed descriptive analyses, including total number, minimum, median, and maximum number of drug switches per month. The pattern of drug switches was visually assessed using a plot of drug switches over time, an overlay plot of total drug switches per year separately, and mathematically by autocorrelation. Autocorrelation was calculated as the number of drug switches in a month, divided by the number of drug switches in a previous month, varying from 1 to 18 months, to identify the lag time in months, which results in the highest correlation.

Furthermore, the number of drug switches is expressed as a percentage of the total number of consecutive dispenses, and the numbers of generic-to-generic (GG), brand-name-to-generic (BG), generic-to-brand-name (GB), and brand-name-to-brand-name (BB) drug switches, as well as the total number of drug switches involving parallel import products, were calculated. In a bar plot, the study period was divided into tertiles to provide a general impression of changes over time. Lastly, drug switches were studied for each active substance, and the analysis for atorvastatin is presented as an example.

We identified the Dutch brand name and EU reference dates from the GIP database [17], the database of the Dutch Medicines Evaluation Board [18], and the EU reference date list [19]. The EU reference date is the earliest EU-known marketing authorization for the (combination of) active substance(s).

### Data management

Data were extracted from the GIP database using SAS Enterprise Guide software (version 7.1). Monthly aggregated data were exported as a Microsoft Excel workbook and imported into R software (version 3.5.0) [20] using the package ‘xlsx’ [21]. Moreover, qualitative and quantitative descriptive analyses as well as data visualization were performed using base R and R Studio [22].

## Results

### Dataset

We identified 23.8 million drug switches on a total of 205.6 million (11.6%) consecutive dispenses. The median percentage drug switches of consecutive dispenses for the included active substances ranged between 15.8% for ethinylestradiol/levonorgestrel and 1.75% for levothyroxine (see Table 1). In some months, for atorvastatin, losartan, pantoprazole, and irbesartan, more than 60% of consecutive dispenses were drug switches.

**Table 1:** Overview of the active substances investigated, with INN, brand name (in the Netherlands), EU reference date, average yearly drug switches, average yearly number of repeat dispenses (not switching), and percentage of drug switches of consecutive dispenses per month (median, range) from June 2009 to December 2016. Active substances are tabulated in descending order by median drug switch percentage

INN	Brand name	EU reference date	Average yearly drug switches	Average yearly repeat dispenses (not switching)	% drug switches median (range per month)
ethinylestradiol/ levonorgestrel	Microgynon®	April 1965	63,824	331,419	15.8% (7.44–48.9)
atorvastatin	Lipitor®	November 1996	264,776	1,297,895	15.0% (4.71–68.1)
salmeterol/ fluticasone	Seretide®	October 1990	104,663	689,450	14.4% (3.10–26.2)
perindopril	Coversyl®	June 1988	161,155	890,803	14.0% (4.31–34.2)
losartan	Cozaar®	September 1994	118,979	735,195	12.0% (3.94–63.6)
simvastatin	Zocor®	April 1988	559,303	3,220,461	11.8% (3.24–47.4)
paroxetine	Seroxat®	December 1990	112,555	754,569	11.8% (4.63–38.2)
pantoprazole	Pantozol®	Augustus 1994	289,171	1,897,439	10.7% (4.06–63.2)
irbesartan	Aprovel®	Augustus 1997	58,854	398,297	9.93% (0.00–60.8)
venlafaxine	Efexor®	September 1993	55,719	416,314	9.61% (5.78–39.9)
metformin	Glucophage®	March 1959	252,557	2,231,914	9.41% (2.82–32.3)
omeprazole	Losec®	April 1987	361,669	2,977,064	9.39% (3.38–28.7)
esomeprazole	Nexium®	March 2000	94,460	761,959	8.98% (4.40–48.6)
methotrexate (immunosuppressant)	Metoject®	July 2003	21,310	171,876	8.70% (4.05–35.8)
enalapril	Renitec®	January 1985	102,387	1,027,850	7.69% (2.82–23.1)
metoprolol	Lopresor®	February 1975	359,131	3,479,555	7.41% (3.58–29.9)
salbutamol	Ventolin®	January 1969	65,047	789,636	7.29% (4.24–13.7)
methylphenidate	Ritalin®	October 1954	29,472	529,279	4.81% (2.59–15.7)
rivastigmine	Exelon®	May 1998	4051	53,243	4.02% (0.00–20.3)
methotrexate (antineoplastic agent)	Ledertrexate®	September 1974	2090	60,219	2.21% (0.00–46.4)
levothyroxine	Thyrax Duotab®	January 1952	66,909	1,252,976	1.75% (0.281–26.0)

The highest number of drug switches in a single month for a single active substance – simvastatin – was 149,497 on a total of 328,184 (46.7%) consecutive dispenses in January 2015. The highest number of drug switches in a single month – January 2016 – for a unique combination of drug products before and drug products after the switch was 41,659, which is 96.6% of all 43,107 atorvastatin drug switches in that month.

### Drug switch pattern

**Fig. 1:**
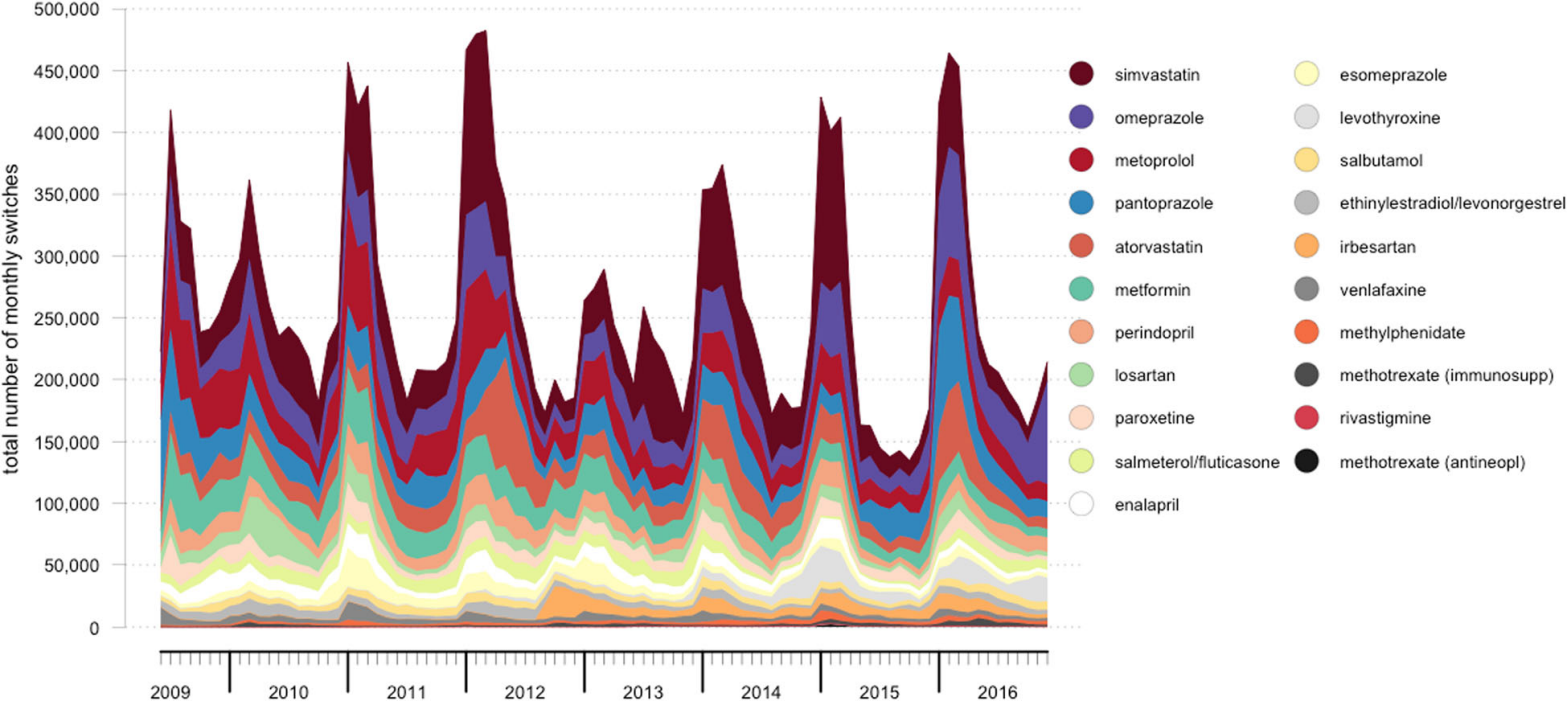
Stacked area chart over time of total number of drug switches per month per active substance from June 2009 to December 2016 in the Netherlands. Active substances are ordered from top to bottom by descending total number of drug switches during the study period. The total number of monthly drug switches is on the y-axis, and the time period of the study is on the x-axis

**Fig. 2:**
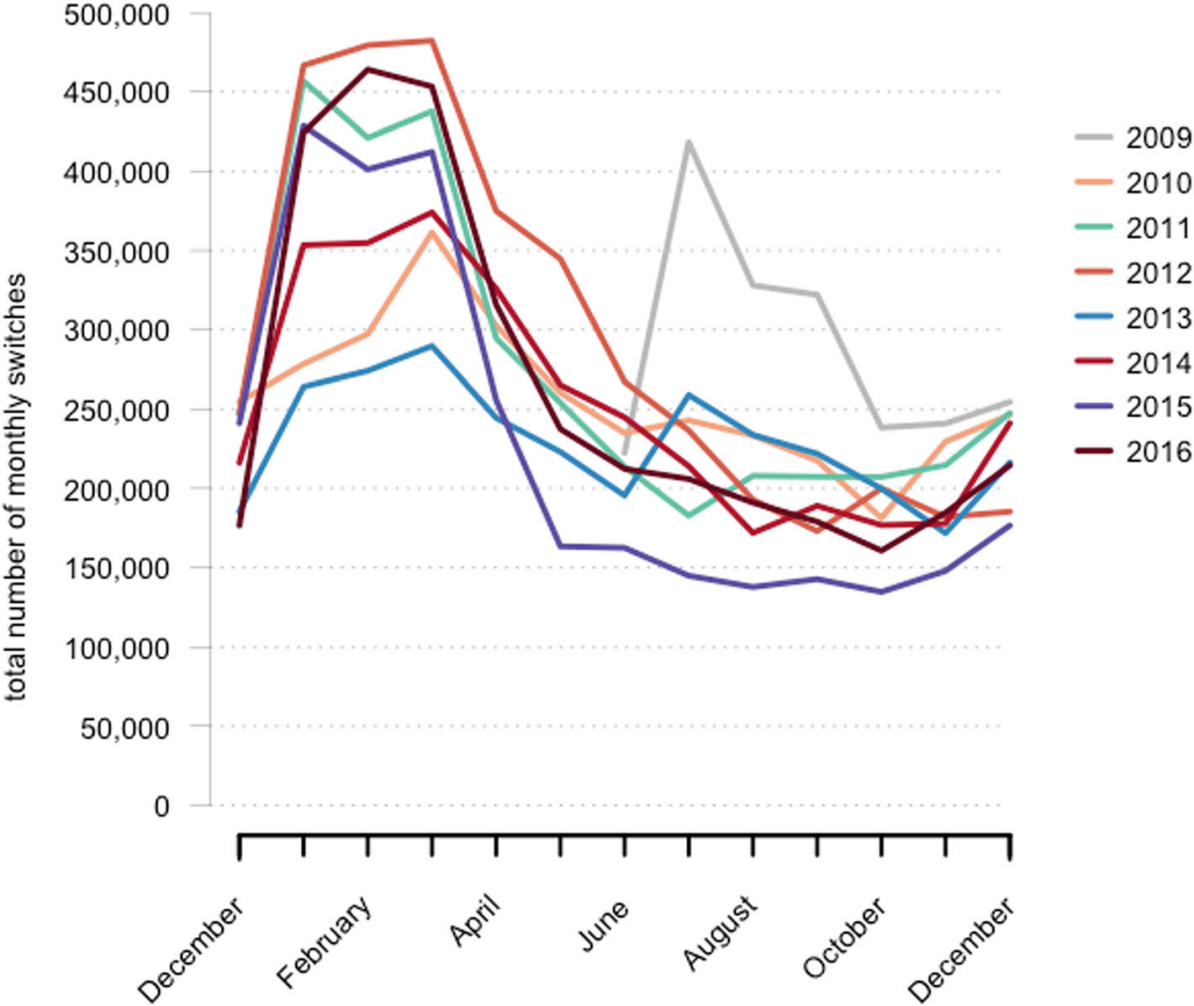
Total number of drug switches per month from June 2009 to December 2016 in the Netherlands for the 20 active substances combined, as an overlay plot in the timeframe of 13 months. Each line represents 13 months of drug switches from December (previous year) to December

An overview of the pattern of drug switches between June 2009 and December 2016 is depicted in Figs. 1 and 2. The pattern of drug switches for the active substances included in this analysis was characterized by seasonality, with most drug switches between January and March of each year between 2010 and 2016. A statically significant positive correlation was observed with a lag of 12 months (autocorrelation = 0.597), which confirms the annual pattern.

### Brand-name drugs versus generic drugs – type of drug switch

**Fig. 3:**
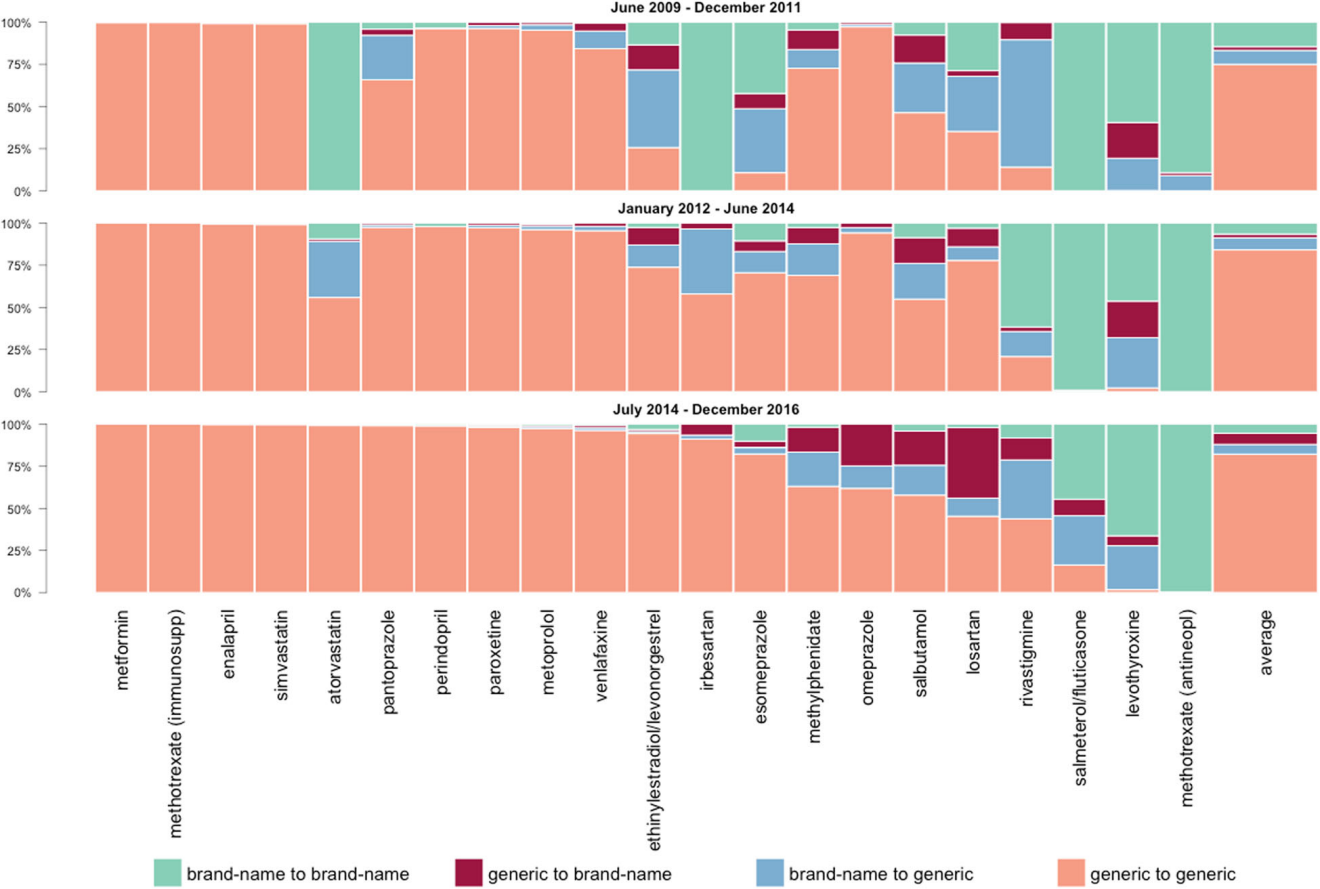
Percentage distribution of type of drug switch per active substance (green: brand-name to brand-name, red: generic to brand-name, blue: brand-name to generic, orange: generic to generic) in descending order by percentage of generic-to-generic drug switch in the last period. Bar plots are (top down) for the periods from June 2009 to December 2011, January 2012 to June 2014, and July 2014 to December 2016

Of all drug switches in our study, only 7.09% (range of active substances 0.00–29.1%) were BG switches. Most drug switches (80.3%, range 0.18–100%) involved a GG switch, while GB switches accounted for approximately 3.52% (range 0.00–17.3%) of drug switches, and 9.06% (range 0.00–99.4%) involved a BB switch of drugs with the same active substance. The distribution of drug switches per drug in tertiles of the time period between June 2009 and December 2016 is illustrated in Fig. 3.

For a number of active substances included in our study, the distribution of the type of drug switch remained relatively stable over the 91 months. However, the type of drug switch involving atorvastatin, irbesartan, esomeprazole, ethinylestradiol/levonorgestrel, salmeterol/fluticasone, omeprazole, losartan, and rivastigmine changed over time. Over these 91 months, atorvastatin, irbesartan, and esomeprazole demonstrated an almost complete change from BB to GG drug switches. For ethinylestradiol/levonorgestrel and salmeterol/fluticasone, the involvement of brand-name drugs in the drug switches reduced as well. In all periods, most omeprazole drug switches were GG, but in the last period, there were GB drug switches (24.7%) and BG drug switches (13.3%) (see Table 2). Furthermore, for losartan, the number of BB and BG drug switches decreased, whereas the proportion of GB drug switches increased to 41.7% between July 2014 and December 2016. For rivastigmine, the proportion of BG drug switches decreased over the three periods (75.6 to 35.0%); however, the proportion of BB drug switches peaked (61.6%) between January 2012 and June 2014 (see Table 2).

**Table 2:** Percentage distribution of type of drug switch per active substance (GG = generic drug to generic drug switch, BG = brand-name drug to generic drug switch, GB = generic drug to brand-name drug switch, and BB = brand-name drug to brand-name drug switch) in descending order by percentage of generic-to-generic drug switch in the last period, similar to the order in Fig. 3. Periods are June 2009 to December 2011, January 2012 to June 2014, and July 2014 to December 2016

	June 2009 to December 2011				January 2012 to June 2014				July 2014 to December 2016			
	GG	BG	GB	BB	GG	BG	GB	BB	GG	BG	GB	BB
metformin	99.9	0.07	0.01	0.00	100	0.00	0.00	0.00	100	0.00	0.00	0.00
methotrexate (immunosuppressive)	100	0.00	0.00	0.00	100	0.00	0.00	0.00	100	0.00	0.00	0.00
enalapril	99.3	0.46	0.27	0.00	99.5	0.32	0.16	0.00	99.7	0.17	0.13	0.00
simvastatin	99.2	0.39	0.38	0.07	99.1	0.27	0.14	0.44	99.7	0.12	0.09	0.09
atorvastatin	0.00	0.00	0.00	100	56.0	33.2	1.34	9.44	99.1	0.50	0.37	0.00
pantoprazole	65.8	26.4	3.64	4.10	97.5	1.46	1.02	0.01	99.1	0.49	0.43	0.03
perindopril	96.4	0.03	0.01	3.54	98.0	0.02	0.00	2.01	99.0	0.05	0.03	0.93
paroxetine	96.3	1.67	1.98	0.00	97.2	1.55	1.23	0.00	97.9	0.74	0.74	0.61
metoprolol	95.3	3.17	1.44	0.06	96.2	2.10	0.99	0.71	97.3	0.91	0.65	1.11
venlafaxine	84.3	10.4	4.88	0.38	95.5	2.61	1.90	0.01	96.2	1.44	1.46	0.91
ethinylestradiol/ levonorgestrel	25.7	46.0	14.9	13.4	73.8	13.2	10.5	2.56	94.3	1.16	1.23	3.31
irbesartan	0.00	0.00	0.00	100	58.1	38.5	3.44	0.00	91.1	2.25	6.59	0.08
esomeprazole	10.8	38.0	9.05	42.2	70.7	12.4	6.34	10.6	82.3	3.77	3.65	10.2
methylphenidate	72.6	11.1	11.7	4.57	69.1	18.5	9.70	2.61	63.1	20.3	14.6	1.90
omeprazole	97.2	1.31	1.37	0.07	94.2	3.11	2.65	0.01	62.0	13.3	24.7	0.07
salbutamol	46.4	29.3	16.6	7.60	55.0	21.1	15.3	8.61	57.9	17.8	20.1	4.24
losartan	35.3	32.6	3.34	28.8	77.8	8.06	11.1	3.01	45.3	10.7	41.7	2.18
rivastigmine	14.0	75.6	10.3	0.08	20.9	14.8	2.67	61.6	43.8	35.0	12.9	8.33
salmeterol/ fluticasone	0.00	0.00	0.00	100	0.00	0.65	0.20	99.1	16.3	29.3	9.78	44.6
levothyroxine	0.15	19.1	21.3	59.4	2.19	29.9	21.7	46.2	1.72	26.1	5.89	66.3
methotrexate (antineoplastic)	0.00	9.04	1.66	89.3	0.00	0.03	0.00	100	0.25	0.02	0.00	99.7
Average	75.0	8.31	2.22	14.5	84.1	7.14	2.18	6.54	82.2	5.59	6.63	5.53

For all active substances combined, the proportion of BB drug switches decreased from 14.5% between June 2009 and December 2011 to 5.53% between July 2014 and December 2016. Conversely, the proportion of GG drug switches increased from 75.0 to 82.2% in the same period.

### Parallel import

A proportion of all drug switches (9.5%) in our study involved a parallel product. These drug switches were mostly BB (81.9%) and BG drug switches (15.8%); however, GB (1.30%) and GG drug switches (1.04%) also occurred. Overall, 0.12% of the GG drug switches and 86.5% of the BB drug switches involved a parallel imported product.

### Detailed data on drug switches – example: atorvastatin

**Fig. 4:**
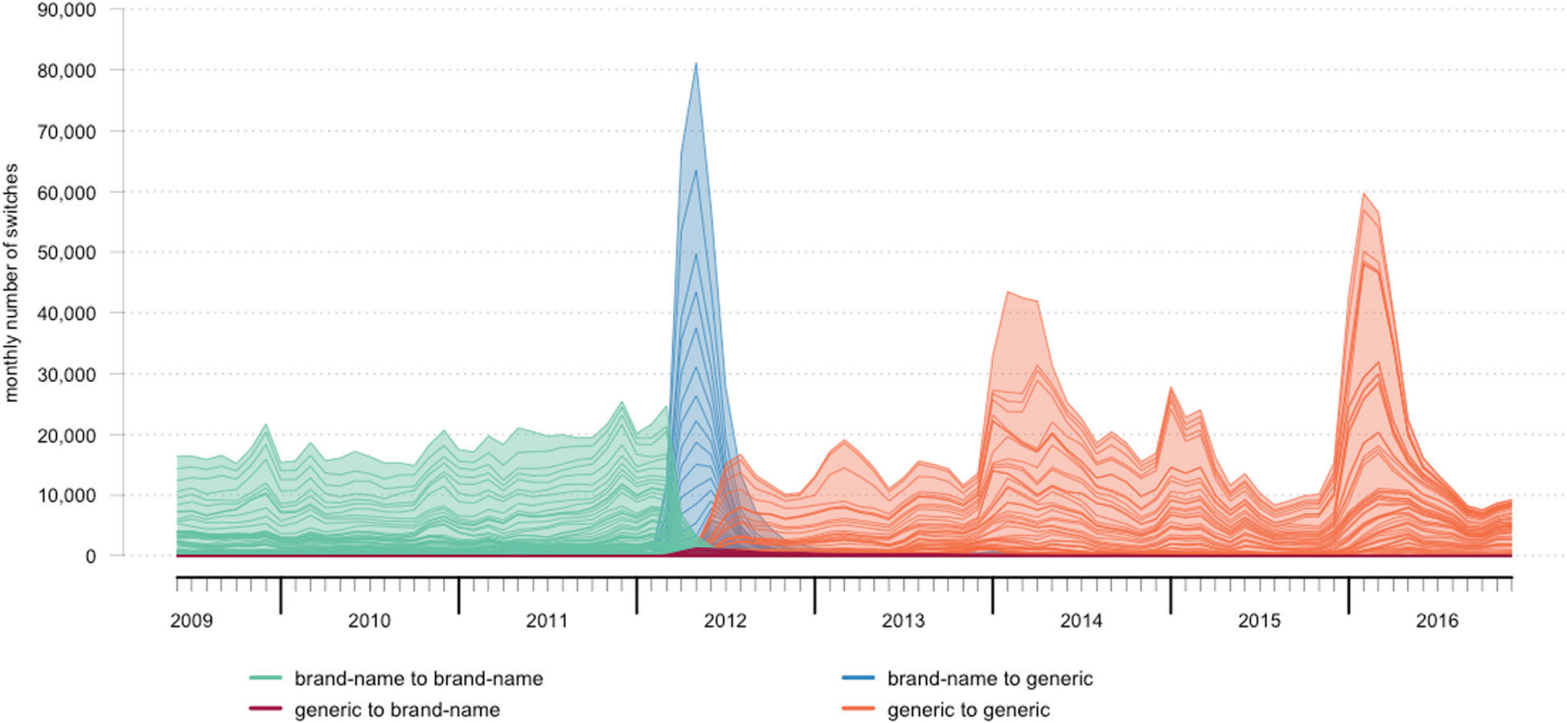
Number of drug switches per month for atorvastatin, color-coded per combination of brand-name or generic drug. Each color is a stacked area plot of the total number of drug switches, separated by solid lines for the individual contribution of switches with a unique combination of drug product before and drug product after the switch

We closely investigated the drug switches of each active substance over time, and we present atorvastatin as an example in Fig. 4 because the patent of Lipitor® expired during our study period (February 2012). Roughly 2 million atorvastatin drug switches were included in our analysis. A large peak in BG drug switches occurred after patent expiry (274,300 BG drug switches in 2012), and only some patients were switched back from brand-name drugs to generic drugs: 1.7% of all drug switches in 2012 (7355/421,875). From July 2012 until the end of the study period, 92.7% of the drug switches (1,057,601/1,141,015) concerned GG drug switches, also exhibiting the annual pattern with a peak between January and March. Moreover, 100% (600,818/600,862) of the drug switches in the period before March 2012 were BB drug switches.

As indicated in Fig. 4, the large BG drug switch peak in 2012 is a summation of 81 different BG drug switches. All atorvastatin switches in our study involved 84 different GG drug switches, 137 BB drug switches, and 68 GB drug switches.

## Discussion

This study provides new insight into the frequency of drug switches in the Netherlands. It is an essential part of characterizing the landscape of generic drug use and an important first step towards elucidating reasons for the occurrence of drug switches.

In our data, an increased number of drug switches were observed in the first 3 months of each calendar year. This increase cannot be explained by a higher overall number of drug dispenses in these months, as we did not observe a change in the number of drug dispenses throughout the year (data not shown). It can also not be explained by the number drug shortages, as these are not known to increase at the beginning of each year. Furthermore, as on average only 4–10% of the Dutch patient population changes health insurance yearly, this cannot explain the increased number of drug switches to a sufficient extent. However, the yearly increase is in line with the typical duration of health insurer-manufacturer contracts and contract renewal at the beginning of each year, as seen in the Preference Policy. Therefore, we postulate that the increased frequency of drug switches in January to March is explained by the Dutch Preference Policy. The deviating pattern of an increased number of drug switches in the second part of 2009, as seen in Fig. 2, is likely caused by the phased introduction of the Preference Policy in that year. Until then, most contracts between manufacturers and insurers were valid for 6 months, whereas from 2009, contracts were usually of longer duration – 1 or 2 years [11].

The yearly pattern of the number of drug switches is most likely explained by the Preference Policy, but only partly. Most Dutch patients are insured by one of four large health insurance companies. Therefore, the expectation would be that there are only four (or less) preferred drug products and thus only a limited number of different drug switches for the same active substance. Nevertheless, as evident from the example of atorvastatin (Fig. 4), peaks in January to March are the result of many different drug switches involving various drug products. This was observed for the other active substances as well (data not shown). Therefore, the influence of other reasons, such as wholesaler and pharmacy practices or both local and international shortages, should be further investigated with the ultimate goal of identifying possible points for improvement towards pharmaceutical care in which the financially wanted market share of generic drugs is large, but the clinically unwanted drug switches do not occur frequently.

If a patient has complaints following the use of, or switch to, a different drug product, then, on medical grounds, a medical doctor in the Netherlands is entitled to prescribe that patient a drug product principally not covered by his or her health insurance (‘medical necessity’ similar to ‘Dispense as Written’ in the US). It is then reimbursed nonetheless and is thus also included in our dataset. Medical necessity is an interesting research topic, as it could be a surrogate for clinical problems or patient satisfaction with the use of a generic drug. In our dataset, we found an average ‘switchback rate’ (generic drug switched to brand-name drug) of 3.52%, with a wide range for the different active substances (0.00–17.3%). However, although a switchback is most likely the result of medical necessity, it could also be caused by other factors, such as supply shortage of the generic drug or a decrease in the price of the brand-name drug. Most importantly, medical necessity is not restricted to a brand-name drug but could also be used to prefer a specific generic drug, which further diffuses the relation between satisfaction and switchback rates. Our dataset is thus not well suited to study clinical discomfort and switchback rates or medical necessity.

Furthermore, we observed a high proportion of BB switches of drugs containing levothyroxine or methotrexate (for the antineoplastic indication) (see Fig. 3). This is most likely explained by the non-availability of generic drugs for these active substances and the availability of parallel imported brand-name drugs.

Over time, for atorvastatin, irbesartan, esomeprazole, ethinylestradiol/levonorgestrel, salmeterol/fluticasone, omeprazole, losartan, and rivastigmine, the type of switch involved fewer brand-name drugs. This observation is most likely explained by the increased availability of generic drugs after patent expiry of the brand-name drug. Indeed, generic drugs of esomeprazole were first available in 2011, those of atorvastatin in 2012, and those of salmeterol/fluticasone in 2013, after which the GG drug switch rate increased.

We only studied drug switches between active substances of the same strength and thus excluded drug switches for which both strength and manufacturer were changed. Although we thereby underestimated the number of drug switches, we believe the volume of these changes is relatively small, and this is not expected to influence our results to a great extent. Moreover, drug switches in which patients were switched between a fixed dose combination (FDC) and a combination of monotherapies were not included. Given the total number of patients for whom FDCs were dispensed in the Netherlands in the GIP database, this could affect drug switches involving perindopril, losartan, irbesartan, and enalapril (all available in FDCs with diuretics). Therefore, this study might underestimate the total number of drug switches for these active substances.

A further limitation is that we investigated only 20 (combinations of) active substances. These 20 were chosen because for these active substances, at least 25 ADRs in relation to drug switching had been reported to Lareb between January 2006 and September 2016 [7]. At a later stage, our drug switch data can be used to refine the Lareb analysis. Since these are the active substances with the highest number of ADR reports related to drug switching, it is possible that we selected active substances that are switched more often than active substances not included in our study. As we do not have drug switch data for other active substances, we can neither confirm nor deny this. However, the 20 active substances represent a wide range of therapeutic classes and include 5 of the 10 most prescribed active substances in the Netherlands (2016). Therefore, we believe that, to a reasonable extent, our study findings can be generalized to other active substances in the Netherlands.

A strength of our analysis is that the drug switch data for each active substance are near complete. The data source, namely, the ZIN GIP, covers approximately 96% of the insured Dutch population [23] and all individuals living or working in the Netherlands are obliged to take out health insurance. Furthermore, the size of or our sample (almost 24 million switches) is large enough for us to be able to draw conclusions about the characteristics of drug switching in the Netherlands with sufficient certainty.

To our knowledge, this is the first nationwide quantitative analysis of drug switches at a patient level in the Netherlands. The amount of literature on this topic is limited. One other study described a quantified approach to generic switching in the Netherlands [24]. However, it focused on the difference between what had been prescribed by the doctor (brand-name drug or generic drug) and what was dispensed by the pharmacist (brand-name drug or generic drug). It did not characterize the frequency of drug switches at the level of the individual patient, as in our study.

Furthermore, a recent FDA study introduced a descriptive tool to analyze novel utilization and drug switching patterns at the manufacturer level [8]. That study focused on the number of new users per manufacturer, time to switch to a generic drug, and switchback rates. Extensive drug switching between drugs produced by different manufacturers was observed, with the exception that there was no distinct annual pattern. In addition, switchback rates could not be compared directly to our study, as these were presented as cumulative incidence rates (close to 20% in 2 years) but seem to be in the same order of magnitude as the rate we observed. In general, our results are consistent with those of the FDA study. The absence of an annual pattern in the American drug switching data suggests that the annual switching pattern we observed is unique to the Netherlands, which is an additional argument that the pattern is probably explained by the Preference Policy.

Although we present data specifically for the Dutch situation, our research has international relevance. Internationally, there is variability with regard to generic market penetration, generic pricing, reimbursement, and the policy for the promotion of generic drugs. Even different reimbursement policies for different types of active substances exist [5, 25–27]. While we postulate a strong influence of a specific Dutch reimbursement policy the drug switch pattern does clarify the downside of a system in which generic drugs are preferred predominantly based on pricing and on frequent changes to the contracts between insurers and manufactures. Especially because of the variability of international policies, other policymakers must face comparable situations, and being aware of the Dutch situation is beneficial for their decision-making process.

## Conclusion

Our results present a unique and extensive characterization of the frequency of drug switches in the Netherlands. We show that for the studied selection of 20 active substances, switching between drugs made by different manufacturers was common during mid-2009 until the end of 2016. Furthermore, we demonstrate that a large number of different drug products are involved in the drug switches, and an increased rate is observed in January to March each year, most likely explained by the Dutch health insurance Preference Policy. Not only are the data valuable as is, but they also serve as a first step towards elucidating the reasons for the occurrence of those drug switches. In addition, the data can be used to put into perspective the absolute number of ADRs associated with drug switching in the Netherlands.

## Data Availability

The data that support the findings of this study are available from the Dutch National Health Care Institute, but restrictions apply to the availability of these data, which were used under license for the current study, and so are not publicly available. Data are however available from the authors upon reasonable request and with permission of the Dutch National Health Care Institute.

## Abbreviations

ADR: Adverse Drug Reaction
ATC: Anatomical Therapeutic Chemical
EU: European Union
FDA: US Food and Drug Administration
FDC: Fixed Dose Combination
GIP: Drug Information System
US: United States
ZIN: National Health Care Institute

## Drug switches

BB: Brand-name-to-Brand-name
BG: Brand-name-to-Generic
GB: Generic-to-Brand-name
GG: Generic-to-Generic
